# Autoencoders for sample size estimation for fully connected neural network classifiers

**DOI:** 10.1038/s41746-022-00728-0

**Published:** 2022-12-13

**Authors:** Faris F. Gulamali, Ashwin S. Sawant, Patricia Kovatch, Benjamin Glicksberg, Alexander Charney, Girish N. Nadkarni, Eric Oermann

**Affiliations:** 1 Icahn School of Medicine, New York, NY 10029 USA; 2 New York University, New York, NY 10016 USA

**Keywords:** Statistics, Epidemiology

## Abstract

Sample size estimation is a crucial step in experimental design but is understudied in the context of deep learning. Currently, estimating the quantity of labeled data needed to train a classifier to a desired performance, is largely based on prior experience with similar models and problems or on untested heuristics. In many supervised machine learning applications, data labeling can be expensive and time-consuming and would benefit from a more rigorous means of estimating labeling requirements. Here, we study the problem of estimating the minimum sample size of labeled training data necessary for training computer vision models as an exemplar for other deep learning problems. We consider the problem of identifying the minimal number of labeled data points to achieve a generalizable representation of the data, a minimum converging sample (MCS). We use autoencoder loss to estimate the MCS for fully connected neural network classifiers. At sample sizes smaller than the MCS estimate, fully connected networks fail to distinguish classes, and at sample sizes above the MCS estimate, generalizability strongly correlates with the loss function of the autoencoder. We provide an easily accessible, code-free, and dataset-agnostic tool to estimate sample sizes for fully connected networks. Taken together, our findings suggest that MCS and convergence estimation are promising methods to guide sample size estimates for data collection and labeling prior to training deep learning models in computer vision.

## Introduction

Supervised learning with deep neural networks has achieved state of the art performance in a diverse range of applications. An adequate number of labeled samples is essential for training these systems but most real-world data is unlabeled. Label generation can be cumbersome, expensive and is a major barrier to the development and testing of such systems [1].

Ideally, when confronted with a task and unlabeled data, one would like to estimate how many examples need to be labeled to train a neural network for that task. In this paper, we take a step towards addressing this problem.

Consider a fully connected neural network *f* of pre-specified dimensions and a dataset *X*, which is initially unlabeled, but for which labels *y* can be obtained when needed. We define the minimum convergence size (MCS) for *f* on *X* to be the smallest number *n* such that a subset *X*_*n*_ of *n* examples drawn at random from *X* can be labeled and used to train *f* as a non-trivial classifier, that is, one whose area-under-the-curve (AUC) on a held-out test set is greater than 0.5:

1 $${{{\rm{MCS}}}}:=\mathop{{{{\rm{arg}}\,{\rm{min}}}}}\limits_{n}(E[{{{\rm{AUC}}}}({f}_{{X}_{n},{y}_{n}}({X}_{test}),{y}_{test})]\, >\, 0.5)$$

Given that outcomes are balanced, an *AUC* > 0.5 implies that a model is able to identify some signal in the underlying data, and if that AUC is on the test dataset, this means that the signal identified by the model can generalize to unseen data. In this scenario, below the MCS, we would expect to see little or no correlation between sample size and model performance measured by AUC, whereas above the MCS we would expect to see a positive correlation.

**Fig. 1:**
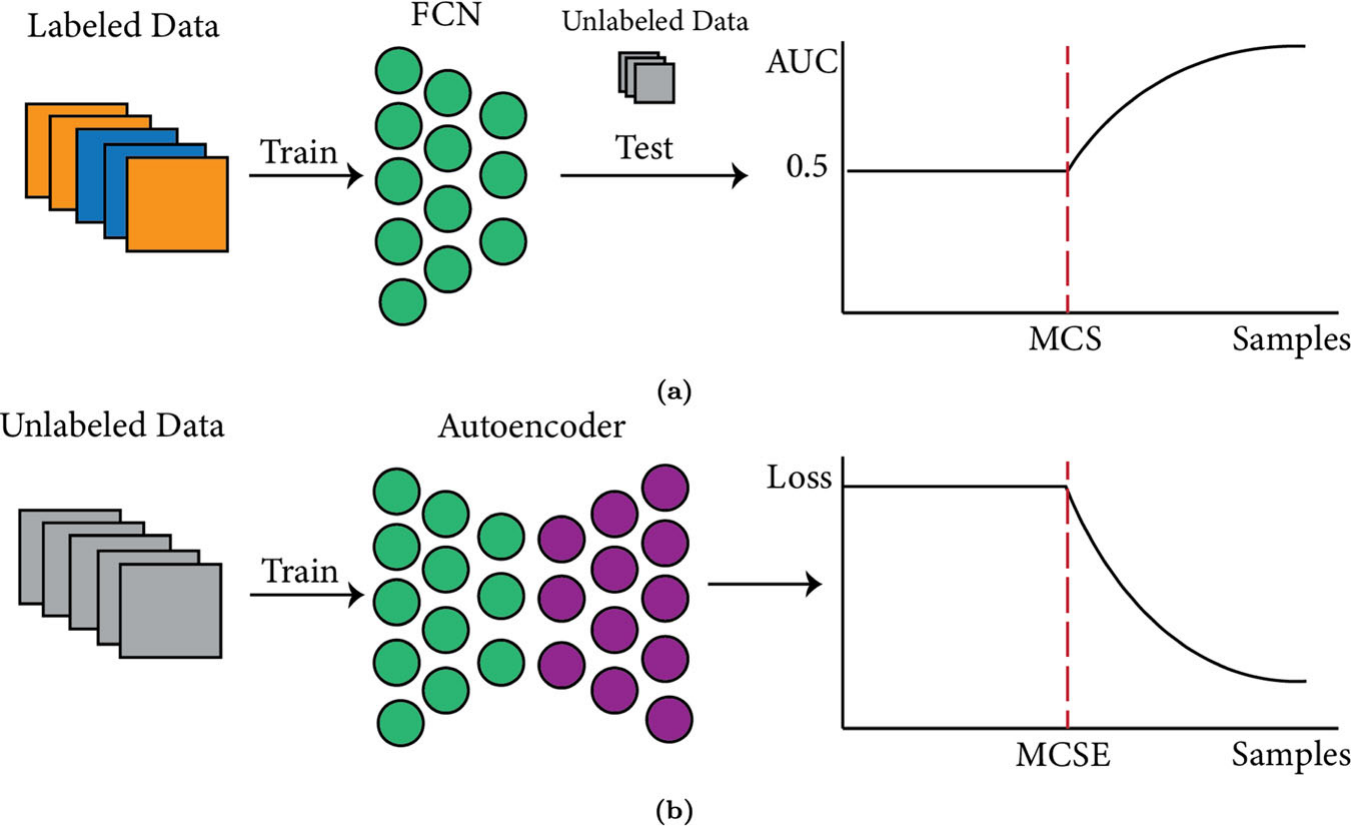
Minimum convergence sample estimation can be used to approximate the number of labels required for generalizable performance. **a** A fully connected network is trained on labeled data, and tested on a unlabeled data. Generalizability Performance is measured via AUC. Minimum convergence sample (MCS) reflects the minimum number of labeled samples required for a fully connected network to start generalizing. **b** An autoencoder with a similar structure as the fully connected network is trained on unlabeled data and the loss function measures how generalizable the FCN is. Minimum convergence sample estimate (MCSE) approximates the minimum convergence sample (MCS).

We propose a method for empirically determining the MCS for *f* on *X* using only unlabeled data, and we call this estimate the Minimum Convergence Sample Estimate (MCSE). We do this by first constructing an autoencoder *g* [2], wherein the encoder part has a similar number of parameters and hidden layers as *f*. We train *g* on increasingly larger (unlabeled) subsets *X*_*i*_ of *X*. This may permit similarities in layer-wise learning between *f* and *g*. Under these circumstances, we empirically show that, at each step *i*, the reconstruction loss *L* of *g* is related to the generalization performance of *f* trained on a similarly sized sample. We also demonstrate how this can be used to determine the MCSE for *f* on *X* (Fig. 1).

**Fig. 2:**
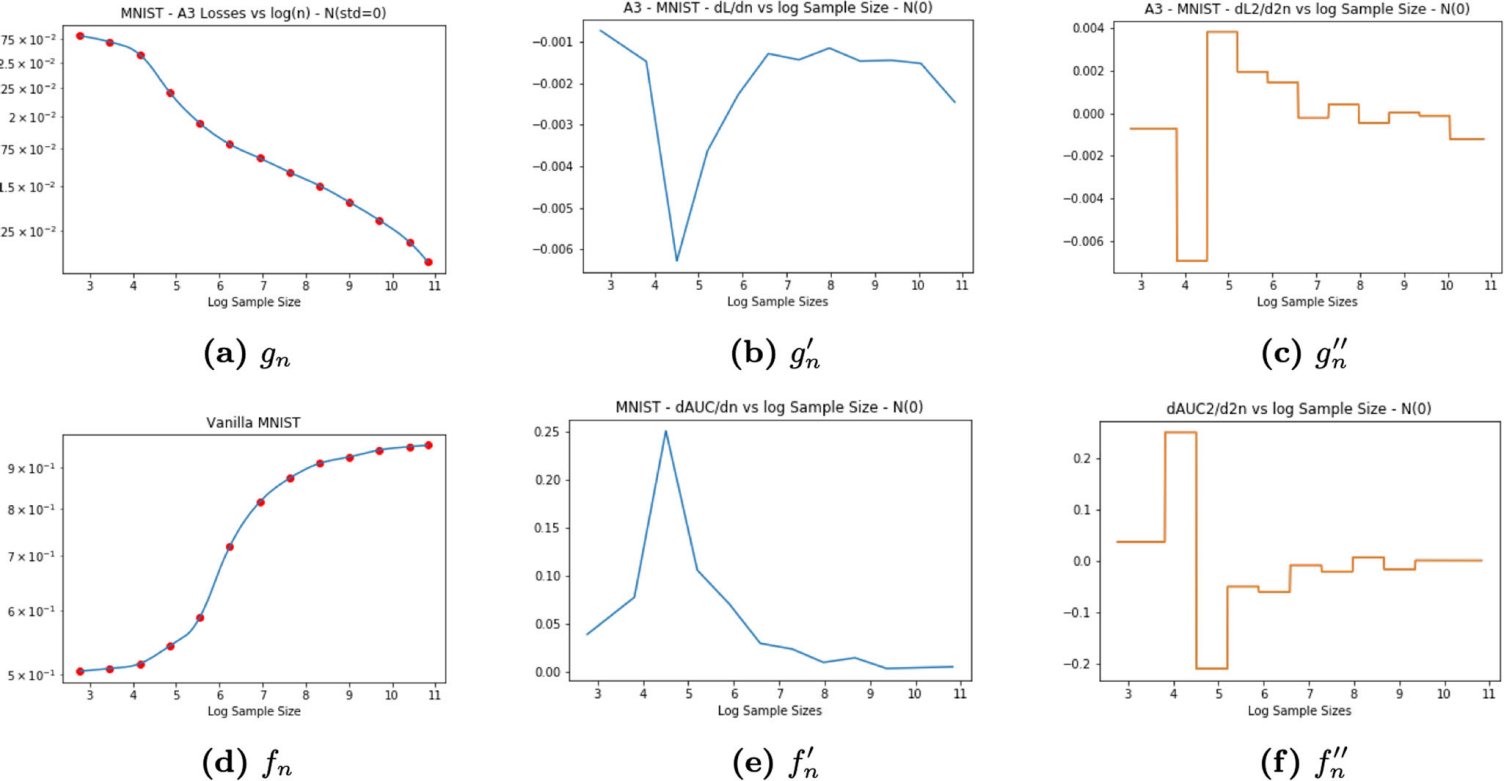
Comparison of autoencoder learning curve with generalizability of a fully connected network. *f* is a fully connected network with input dimension of 784 and output dimension 10, and *g* an autoencoder with an input dimension of 784 and a latent space of dimension 3. **a** The loss of the autoencoder displays a curve split into two phases: the quick phase and the slow phase. **b** The first derivative of the autoencoder loss function displays a decay phase and a growth phase. **c** The second derivative reveals a sharp inflection point where the slope changes from sharply decreasing to sharply increasing. **d** The area-under-the-curve metric on the test set displays a biphasic structure: a rapid growth phase and a slow growth phase. **e** The first derivative of the AUC curve reveals a rapidly increasing phase followed by a decreasing phase. **f** The second derivative of the AUC curve reveals an inflection point as a mirror image of the autoencoder loss curve.

As an example, consider classification of the MNIST [3] dataset with a fully connected neural network (Fig. 2). A comparison of the test set AUC curve of *f* and the loss curve of autoencoder *g* shows that their inflection points occur at similar sample sizes. We then define the MCSE for *f* on MNIST as the sample size corresponding to the inflection point in the loss function of *g*:

2 $$\begin{array}{lll}{{{\rm{MCSE}}}}&:=&\mathop{{{{\rm{arg}}\,{\rm{max}}}}}\limits_{n}\frac{{{{{\rm{d}}}}}^{2}L}{{{{\rm{d}}}}{n}^{2}}\\ &\approx &4.5\,\,({{{\rm{in}}}}\,{{{\rm{this}}}}\,{{{\rm{case}}}})\end{array}$$

With sample sizes above MCSE, the learnability of the dataset on *f* may be approximated by the ease with which *g* is able to embed a latent space that fully represents the data. We hypothesized the following relationship between generalization power of a classifier with respect to learnability of the dataset by the corresponding autoencoder:

3 $$\frac{{{{\rm{d}}}}{{{\rm{AUC}}}}}{{{{\rm{d}}}}n}{{{\rm{AUC}}}}({f}_{n})\simeq \left\{\begin{array}{ll}0,&{{{\rm{for}}}}\,n \,<\, {{{\rm{MCSE}}}}\\ -\beta \frac{{{{\rm{d}}}}L}{{{{\rm{d}}}}n}L({g}_{n}),&{{{\rm{for}}}}\,n\ge {{{\rm{MCSE}}}}\\ \end{array}\right\}$$

*β* is a scaling constant. We tested this hypothesis by calculating the correlation coefficient below the MCSE and above the MCSE, and results are reported in Table 1. A significant *R*^2^ indicates a linear correlation between loss and power. We used eight different standard computer vision datasets to demonstrate our method. MNIST, EMNIST [4], QMNIST [5], KMINST [6] are character-recognition datasets composed of 28x28 pixel grayscale images. FMNIST[7] contains 28x28 pixel grayscale images of objects. CIFAR-10 [8] contains 32x32 pixel color images. STL-10 [9] contains 96x96 pixel color images with fewer labeled images than CIFAR-10, and many unlabeled images. FAKE is a dataset of randomly generated images in PyTorch. We also extended our study to synthetic data and a publicly available medical imaging dataset.

**Table 1:** Comparison of statistical power above and below auto-encoder loss inflection points for various image datasets.

	Pre-MCSE			Post-MCSE		
Dataset	*R*^2^	Kendall’s *τ*	Spearman’s *ρ*	*R*^2^	Kendall’s *τ*	Spearman’s *ρ*
MNIST	0.449	0.438	0.611	0.751	0.851	0.972
FMNIST	0.203	0.286	0.411	0.922	0.855	0.969
KMNIST	0.129	0.284	0.414	0.919	0.839	0.971
EMNIST	0.119	0.224	0.336	0.784	0.843	0.971
QMNIST	0.207	0.323	0.476	0.779	0.836	0.962
CIFAR10	0.028	0.051	0.073	0.499	0.777	0.936
STL10	0.002	0.013	0.016	0.002	0.144	0.209
FAKE	0.002	0.085	0.128	0.154	0.032	0.046

## Results

### Image data results

**Fig. 3:**
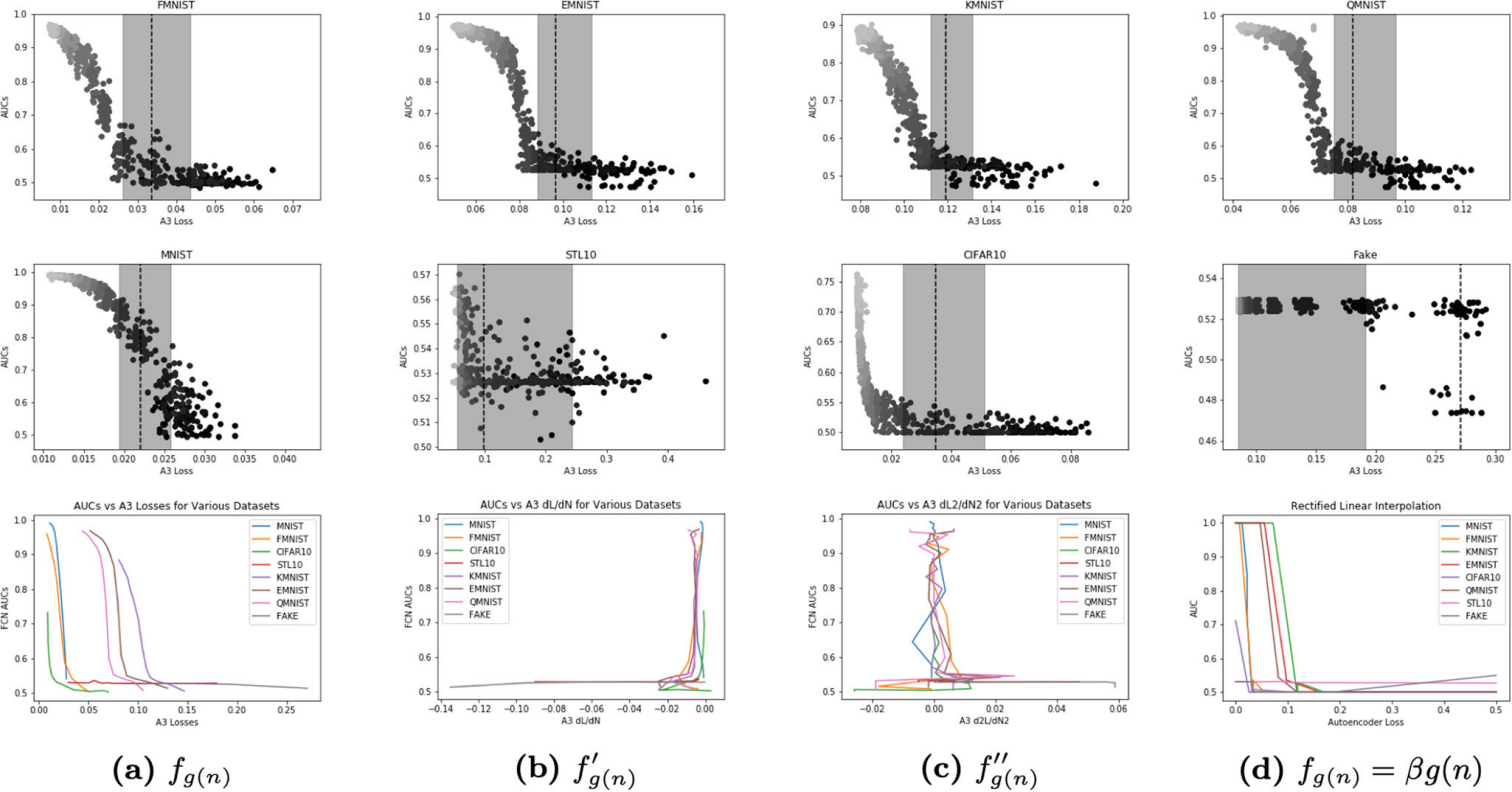
Sample size estimation with inflection points. The top and middle rows are the results of individual datasets, while the bottom row is the combination of all eight tested datasets. The black striped line represents the autoencoder loss at the point of the inflection. The shaded region represents the error bars, with error determined as the autoencoder loss at ± ln(n), where n is the sample size at which the inflection point of the autoencoder loss occurs. The points are shaded by sample size. For each of these datasets, the autoencoder loss method appears to provide an unbiased estimate of the minimum convergence sample. The top row demonstrates appropriately sampled data while the middle row shows statistical power estimation on oversampled and undersampled data. The bottom row shows that linear interpolation using auto-encoder loss function generally works well in estimating learnability. **a** Test Area-Under-the-Curve Metric as a function of autoencoder loss. **b** Test Area-Under-the-Curve Metric as a function of the derivative of the autoencoder loss. **c** Test Area-Under-the-Curve metric as a function of the double derivative of the autoencoder loss. **d** Linear interpolation of autoencoder loss with respect to Test Area-Under-the-Curve metric.

Our autoencoder-based method estimates the inflection point of the fully connected network’s loss function, and therefore provides an effective measure of the minimum sample size required for the network to learn meaningful representations and become statistically powerful for the FMNIST, EMNIST, QMNIST, CIFAR10, and STL10 datasets (Fig. 3). This technique has the potential to be highly useful for providing a priori estimates of the amount of labeled data that must be gathered at the onset of a project. In medical, biological, military intelligence, and other applications where substantial and costly domain expertise is required for labeling data, our empirical method can provide a valuable alternative to relying on subjective experience—the current state of the art for ML sample size estimation.

The two main variants from our experiment were CIFAR-10, which has a larger input dimension, and STL-10, which has a smaller sample size. Although the CIFAR-10 dataset had a larger input dimension compared to the other datasets, the results were valid and interpretable, which suggests that this autoencoder method can be generalized across input sizes. Furthermore, we held the number of classes constant at ten for ease of interpretation, but the observed results hold for different label sets. Second, STL-10 had far fewer samples than the NIST datasets, suggesting that this method for estimating the minimum learnable sample size may be used on datasets with small sample sizes.

The fully connected network demonstrates a growth in statistical power above the compressibility on the MNIST dataset (Fig. 3). This may be due to the unknown and arbitrary data pre-processing steps conducted on the MNIST dataset [10], which allow a fewer number of samples for classification than compression, biasing the autoencoder’s learnable sample size estimate downward. However, it is reassuring that the fake data leads to autoencoder loss instability, resulting in error bars that exist out of the bounds of the estimate. This estimate suggests that the provided fully connected network would not be able to decipher a distribution from the fake data, which places a bound on the learnability of the dataset with respect to the network.

Through non-parametric correlation tests, we showed that loss and power are uncorrelated before the minimum convergence sample estimate, and that they are correlated above the minimum convergence sample estimate (Table 1). The high *R*^2^ values on datasets such as FMNIST and KMNIST validate the linear relationship between classifier power and autoencoder loss in Eq. (3). However, the higher values of Kendall’s *τ* and Spearman’s *ρ*, which both measure non-linear correlation, suggest that in some cases there is potentially a third stage of learning beyond compression. This may be related to the regularization of parameter weights or fine tuning of the network to ensure labels are semantically linked to their ground truth representation. Nevertheless, Kendall’s *τ* and Spearman’s *ρ* are able to fully capture these non-linear trends, even with extremely small sample sizes, as demonstrated with the STL-10 dataset, or with larger networks, as demonstrated with the CIFAR-10 dataset.

**Fig. 4:**
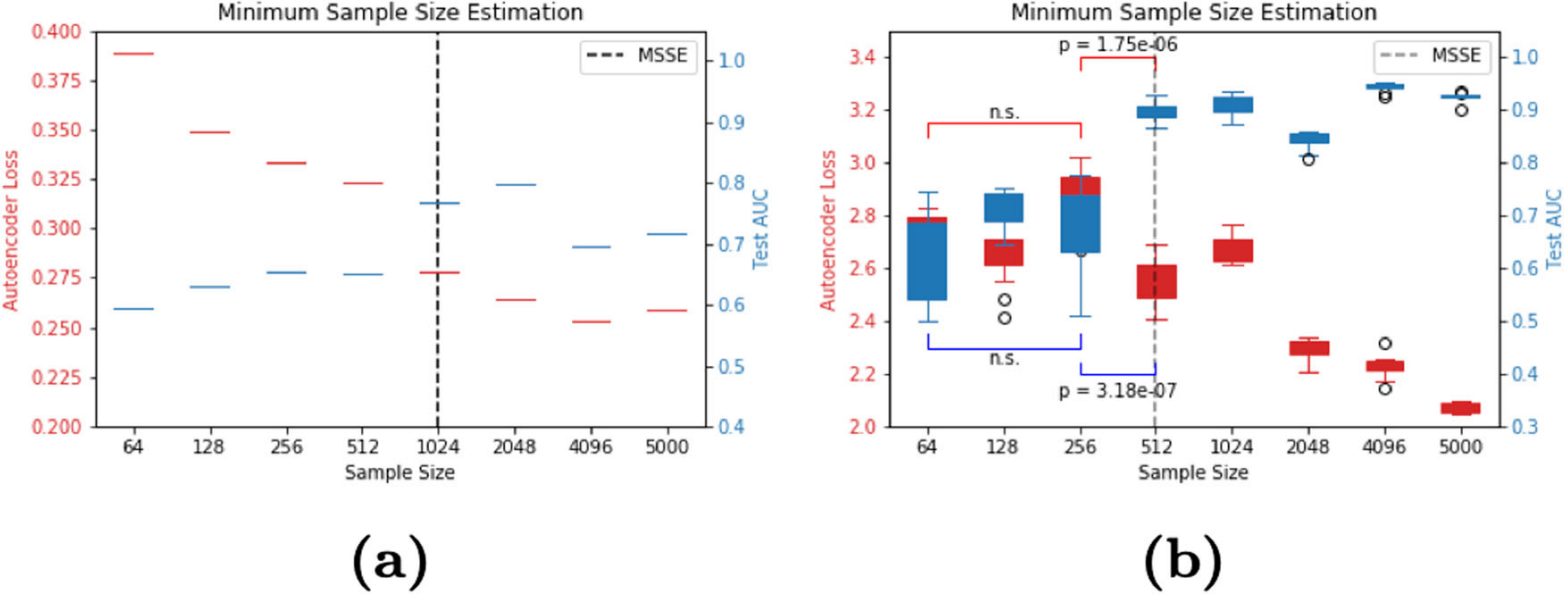
Minimum convergence sample estimation on a medical imaging and synthetic datasets. **a** Minimum convergence sample estimation on a medical imaging dataset for pneumonia detection on Chest X-rays. **b** Minimum convergence sample estimation on a synthetic dataset (×10 bootstrapped). Boxes represent the 25th and 75th percentiles, and the whiskers and the whiskers represent the minimum and maximum values.

Finally, we demonstrate that this method works specifically for medical imaging datasets, where labeling is especially time-intensive and costly, and more generally on a synthetic datasets (Fig. 4a). We use a medical dataset consisting of X-ray images for pneumonia detection and show that the AE accurately predicts a minimum convergence sample at *n* = 1024 samples. We anticipate sample size estimation will reduce the dataset burden for medical imaging tasks.

### Synthetic data

To generalize our work more broadly to generic clustered representations, we demonstrate that this method works on synthetic data. We randomly sampled data-points from an n-dimensional hyper-cube with side-lengths equal to the class separation to train the fully connected network (FCN) classifier and autoencoder (Fig. 4b). The minimum convergence sample estimate, which is the sample size at which the autoencoder loss (red) first shows a significant improvement, occurs at *n* = 512 samples. Examination of the FCN classifier AUCs shows that no significant learning occurs until the sample size reaches the minimum convergence sample estimate of *n* = 512, at which point a significant improvement in AUC is observed.

We run the minimum convergence sample estimator across synthetic data, varying the number of input features, the number of classes, the number of informative features, and the number of hidden units. We find that MCSE invariantly captures the number of samples required for the fully connected network to generalize to an unseen dataset (Table 2).

**Table 2:** Correlation between test area-under-the-curve and sample size above and below auto-encoder loss inflection points for with varying dataset characteristics and neural network hyper-parameters.

		Pre-MCSE			Post-MCSE		
Parameter	Value	*R*^2^	Kendall’s *τ*	Spearman’s *ρ*	*R*^2^	Kendall’s *τ*	Spearman’s *ρ*
N-informative	8	0.074	0.292	0.341	0.577	0.837	0.944
	16	0.019	−0.172	−0.210	0.578	0.836	0.944
	32	0.013	−0.180	−0.224	0.534	0.850	0.954
	64	0.078	0.172	0.212	0.529	0.835	0.944
	128	0.056	0.138	0.170	0.632	0.804	0.925
	256	0.010	0.081	0.105	0.729	0.707	0.852
N-classes	2	0.000	0.000	0.000	0.161	0.399	0.530
	4	0.011	0.072	0.093	0.332	0.514	0.679
	6	0.039	0.146	0.189	0.522	0.610	0.783
	8	0.029	0.125	0.162	0.615	0.659	0.824
	10	0.023	0.118	0.152	0.606	0.617	0.781
N-features	256	0.004	0.059	0.053	0.681	0.585	0.736
	512	0.149	0.410	0.538	0.666	0.663	0.814
	784	0.020	−0.108	−0.141	0.713	0.697	0.849
	1024	0.225	0.402	0.475	0.566	0.624	0.789
	2048	0.100	0.193	0.253	0.629	0.608	0.769
	4096	0.069	0.181	0.225	0.582	0.513	0.671
Hidden Layer Size	64	0.012	0.085	0.110	0.459	0.499	0.653
	128	0.023	0.118	0.153	0.552	0.591	0.755
	256	0.014	0.085	0.111	0.590	0.619	0.783
	512	0.023	0.109	0.142	0.621	0.638	0.800
	784	0.026	0.123	0.160	0.603	0.656	0.818
	1024	0.039	0.151	0.194	0.595	0.640	0.805

**Fig. 5:**
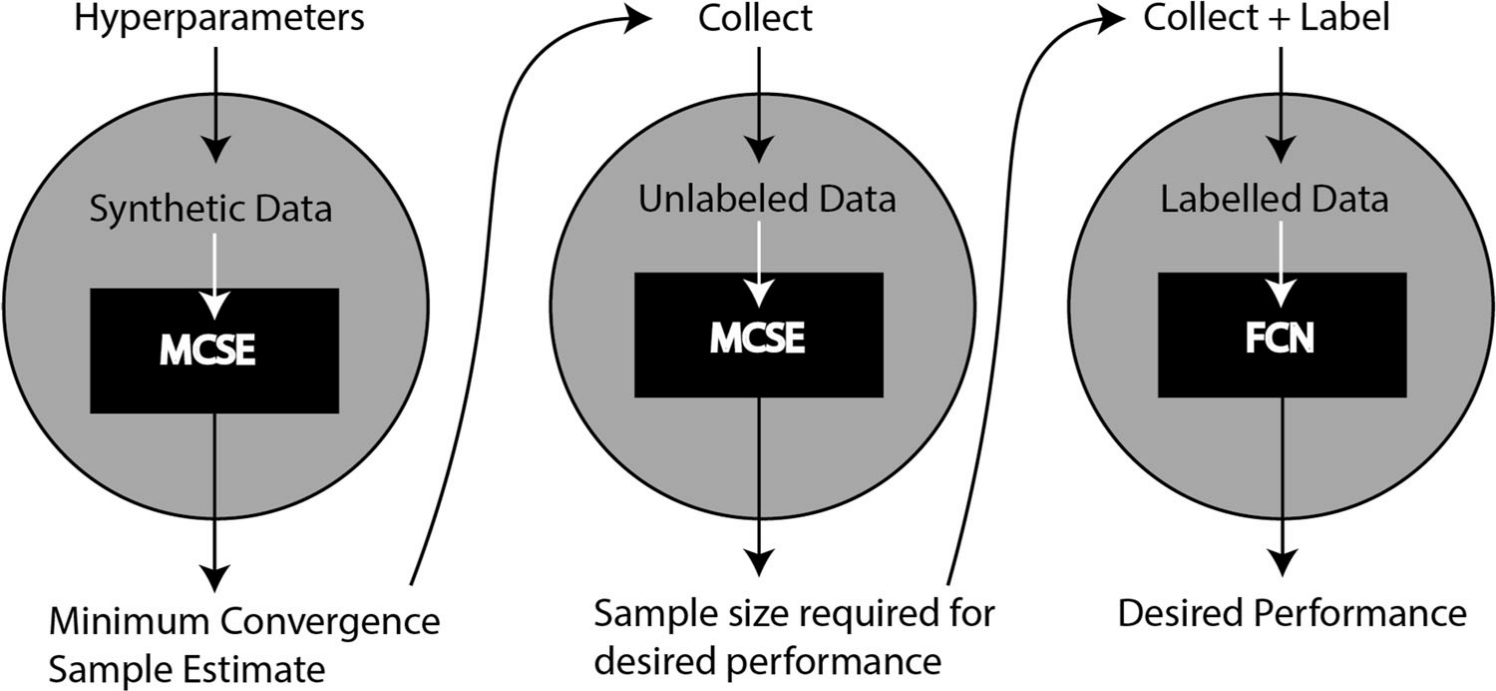
Data collection pipeline with minimum convergence sample estimation. Stage 1 of the pipeline is to use hyper-parameters to estimate a minimum convergence sample. Stage 2 is to collect the number of samples estimated by the MCSE, and use that to determine the sample size required for a desired performance via MCSE and Eq. (3). Stage 3 is to collect the number of samples required, label those samples and train on the FCN to achieve the desired performance.

Because the model works on synthetic data, we are able to provide a code-free, dataset-agnostic tool for researchers to a priori estimate the minimum number of labeled samples they need to train an effective fully connected network classifier. We deploy the application using Flask and PyTorch on a publicly accessible server. We ask for inputs such as priors on the minimum sample size estimate, the number of total features, informative features and classes. The algorithm can be bootstrapped to reduce error, and the size of each hidden layer in a 3-layer fully connected network can be modified as well, to accommodate for dataset complexity. This tool can be easily accessed at samplesizeforml.com. This tool is intended to be used a priori to data collection. Estimating minimum convergence sample on a synthetic dataset can provide a lower bound on the number of samples to collect. After collecting the minimum convergence sample, MCSE can be more precisely estimated and extrapolated via Eq. (3) to obtain an estimate of the number of samples required to achieve a desired performance (Fig. 5).

### Limitations on label quality

Minimum convergence sample estimation, by design, does not account for label quality. Label quality is an essential part of experimental design, and past work has shown that label errors can destabilize bench-marking [11]. For minimum convergence sample estimation to provide a reasonable estimate of minimum convergence sample, it relies on labeled data providing semantically meaningful information.

In situations where label quality does not semantically capture meaningful information in the data, the minimum convergence sample estimate may not provide an accurate estimate of minimum convergence sample. Running the method on fake data generates non-interpretable results (Fig. 3d). The converse is also true, if an autoencoder converges while the fully connected network does not, this may serve as a warning on label and sample quality. To further quantitatively evaluate how label quality affects the minimum convergence sample estimate, we evaluate the minimum convergence sample estimate on synthetic data mislabeled a certain percentage of the time ranging from 0.1 to 50% (Table 3). We demonstrate that as label quality decreases, the MCSE becomes a worse estimator of MCS.

**Table 3:** Correlation between test area-under-the-curve and sample size above and below auto-encoder loss inflection points for with varying levels of label accuracy.

	Pre-MCSE			Post-MCSE		
% Mislabled	*R*^2^	Kendall’s *τ*	Spearman’s *ρ*	*R*^2^	Kendall’s *τ*	Spearman’s *ρ*
0.10	0.006	0.074	0.092	0.686	0.703	0.842
1.00	0.003	–0.025	–0.032	0.710	0.721	0.865
2.00	0.021	0.100	0.126	0.706	0.694	0.839
5.00	0.002	0.054	0.070	0.687	0.654	0.812
10.0	0.001	0.002	−0.001	0.714	0.681	0.827
20.0	0.012	0.084	0.106	0.644	0.619	0.768
30.0	0.004	0.028	0.038	0.465	0.498	0.636
40.0	0.000	−0.017	−0.022	0.389	0.418	0.557
50.0	0.024	−0.105	−0.137	0.385	0.354	0.471

While most standard imaging data-sets have reasonable connections between label and data, more complex data-sets may have a weaker link. This is, therefore, a limitation of present work. A joint pipeline with other methods to ensure label quality provides a direction for future work [12, 13].

## Discussion

Data collection is a universal step in the development of machine learning algorithms. The question of how much labeled data is needed to train a generalizable classifier is one that every data scientist working in supervised or semi-supervised learning must grapple with. The paradigm of big data answers with ‘as much data as we can get’. For many tasks, however, this convention is highly problematic. A priori sample size determination is a common practice for almost every field to mitigate some of these issues, and here we introduce a variant on sample size determination—the minimum convergence sample for machine learning. We additionally propose and validate a method to estimate a minimum convergence sample for deep learning algorithms with a focus on fully connected networks. This study makes several contributions to the field. The first is a simple method for estimating dataset learnability by a given model *f*. While prior work has characterized different methodologies by which learnability can be characterized, these works have focused on characterizing the expressivity of models relative to data rather than the learnability of data relative to a model[14, 15]. Du and colleagues [16, 17] have adapted tools from empirical process theory [18] for the estimation of sample complexity of CNNs and RNNs. However, empirical process theory may not extend well to networks which include nonlinear activation methods, like Rectified Linear Units.

In this work, we primarily focus on estimating the number of labeled samples needed to train fully connected networks. Many developments in deep learning have focused on reducing the number of samples. These approaches fall into two categories: (a) adding structural information about the data into the model or (b) having the model assign labels through semi-supervised learning approaches. An improvement in sample efficiency does not void sample determination, but rather increases the potential gap between the minimum convergence sample and number of collected labeled samples.

In the first category, improvements neural network architectures that take advantage of domain-specific and data-specific knowledge can reduce the minimum convergence samples. For example, neural network layers like convolutions takes advantage of spatial relationships of the pixels in an image to better learn representations of an image [19]. Convolutions improve sample efficiency and achieve generalizable performance at smaller samples than fully connected networks. The technique of a priori minimum convergence sample estimation we present should be easily adaptable to architectures like convolutional neural networks.

In the second category, semi-supervised learning methods can learn labels given a large set of unlabeled samples and smaller subset of labeled samples [20]. Semi-supervised learning relies on a few key assumptions, the most relevant of which is the low-density assumption. The low-density assumption in semi-supervised learning is that the decision boundary of a classifier should preferably pass through low-density regions in the input space. When a dataset is not representative of the population due to systemic inequities as seen in healthcare, this may result in the classifier providing biased labels for underrepresented classes. For example, there may be biases in data collection based upon race and ethnicity because of inequitable access to care [21]. A minimum convergence sample estimate may help guide data collection to adequately represent low-density subsets of the data.

Sample size determination and statistical power remain closely related for many important studies, including medical trials and social and psychological studies [22, 23]. Not having a priori sample size estimation has been recognized as a common mistake in the design of clinical trials and occurs more often when statisticians are not involved early in the trial design process [24]. Under-powered studies in neuroscience, and more specifically in Alzheimer’s disease have led to routine failures in replication [25–27]. Given the increasing presence of artificial intelligence in medicine and clinical trials [28], sample size determination for neural networks represents an important opportunity to advance the utility of artificial intelligence in these domains by increasing trial efficiency, efficacy, and power. Most grant applications in medicine require an estimate of sample sizes, and now an estimate can be reasonably provided for machine-learning based grant applications via the deployed Flask application. A sound method for conducting sample size estimation for machine learning models can ensure proper experiment design.

Some past work has surveyed the use of sample size determination in machine learning with respect to medical imaging applications [29]. These methods are split into Pre-Hoc and Post-Hoc methods. However, pre-hoc methods were not robust in the high-dimensional setting with large intraclass variability [30]. Other pre-hoc methods such as empirical process theory did not extend well to non-linear methods [16]. Post-hoc methods usually involve fitting a learning curve, but fitting a learning curve is trivial for minimum convergence sample estimation because any amount of data should result in a non-zero increase in performance on a training data-set. Moreover, these methods are task-specific, data-specific, and model-specific, as one learning curve has no relevance outside that specific task, model and data-set. Nevertheless, while our experiments validate minimum convergence sample estimation on toy data-sets, synthetic data, and one real-world example of medical imaging due to data availability, future work should further validate this method on more across different tasks and imaging types in the healthcare context.

Our second contribution is the proposal of a method to empirically estimate MCSE for a given fully connected neural network *f*. This function allows users to predict statistical power of a model without needing to train on the entire training set during every trial. It also includes an uncertainty on the estimate, in which the variance is inversely correlated to how structured the underlying data is. Our third contribution is a publicly available tool for minimum sample size estimation for fully connected neural networks.

Importantly, there are several natural opportunities to extend our work to more complex models, as discussed below. First, our paper only considered a fully connected network with a relatively simple architecture. One natural question that might extend from this work involves assessing how this method fares in estimating the statistical power of convolutional or recurrent neural networks. While adding convolutions would be relatively easy to do via the addition of another layer, adding attention mechanisms may require additional structural modifications to fully approximate the statistical power of recurrent neural network or transformers. For our method to be applicable to medical imaging tasks, we anticipate that extending this work to convolutional neural networks remains an important next step. Future work can validate MCSE on more complex architectures utilizing pre-trained networks and skip connections. Second, the loss function that was utilized in this current analysis was the reconstruction loss, which is a relatively simple choice of loss function. For variational autoencoders, the loss function changes to instead use a KL-divergence, while GANs use JS-divergence and WGANs use Wasserstein divergence [31–33]. Therefore, different autoencoders with various structural representations can also be used to represent a fully connected network with distinct losses and structural features. Future work should examine different reconstruction frameworks to approximate the statistical power of increasingly complex network architectures. Third, we have not explored the utility of a similar approach to aid in architecture search and identification of an optimal set of cases for labeling.

In summary, we present a novel method of estimating the minimum sample size required to train a fully connected neural network for a classification task. The distinguishing feature of our approach is that this estimate can be obtained prior to labeling any data, which can be advantageous in real-world settings where labeling is expensive or time-consuming.

## Methods

### Main

For the six MNIST variants and FAKE, we let *f* be a fully connected network with input dimension of 784 and output dimension 10, and we let *g* be an autoencoder with an input dimension of 784 and a latent space of dimension 3. The latent space dimension of 3 was chosen in the hope of avoiding over- or under-compression of the latent space. The only differences for CIFAR-10 and STL-10 were input dimensions of 1024 and 9216, respectively. We train both *f* and *g* on 13 different sample sizes at factors of ×2: 16, 32, 64, 128, 256, 512, 1024, 2048, 4096, 8192, 16,384, 32,768, and 50,000. Training was performed by bootstrapping 50 times on two NVIDIA GeForce GTX 1080 Ti GPUs using PyTorch. We tested all 50 bootstrapped models of *f* at different sample sizes on the test-size of 10,000 to find the test AUC. This protocol was completed on all eight datasets.

Then, we smoothed both the autoencoder loss function of *g* and the area-under-the-curve of *f* using a natural spline. Next, we obtained the second derivative of the spline on the autoencoder (Fig. 3).

After taking the second derivative of the loss function, we located the inflection point and its respective sample size as well as the value of the autoencoder loss at that value. Figure 2 was generated when we plotted the autoencoder loss of *g* and the AUC of *f* against the sample size. *MCSE* was drawn as a vertical line in Fig. 3, coinciding with the inflection point on the autoencoder loss function and provides a lower bound on the sample size required to improve model performance. The shaded area bars represent the error, determined as the autoencoder loss at the *log*(*MCSE* ± 1) sample sizes.

Third, we determined the correlation between autoencoder loss and area-under-the-curve using *R*^2^, Kendall’s *τ* and Spearman’s *ρ* (Table 1). These values demonstrated a significant coorelation above, but not below, the MCSE (inflection point of the autoencoder loss curve). To better demonstrate this finding, we plotted out the results for Eq. (3) for values above *MCSE* (Fig. 3).

Finally, we generalize these results to an n-dimensional hyper-cube and validate them on a medical imaging dataset. To generate the synthetic data set, we randomly sampled data-points from an n-dimensional hyper-cube with side-lengths equal to the class separation to generate the fully connected network classifier and an autoencoder. For the medical dataset, we use the publicly available Kaggle Chest X-ray dataset [34], and accurately predict the minimum number of labeled samples required to learn a meaningful classifier using a fully connected network.

Analysis of the publicly available NIH CXR dataset was carried out with approval of the Institutional Review Board at Icahn School of Medicine at Mount Sinai, New York, NY 10019. The requirement for informed consent was waived as the dataset was completely de-identified.

### Reporting summary

Further information on research design is available in the Nature Research Reporting Summary linked to this article.

## Supplementary information

Reporting Summary

## Data Availability

The image datasets can be accessed via the torchvision library https://pytorch.org/vision/stable/datasets.html. The synthetic dataset was generated via the sklearn library https://scikit-learn.org/stable/index.html. The de-identified X-ray dataset is publicly available https://www.kaggle.com/paultimothymooney/chest-xray-pneumonia.

## References

[1] Sambasivan, N. et al. "everyone wants to do the model work, not the data work”: Data cascades in high-stakes ai. In *Proceedings of the 2021 CHI Conference on Human Factors in Computing Systems*, CHI ’21 (Association for Computing Machinery, New York, NY, USA, 2021).

[2] Goodfellow, I., Bengio, Y. & Courville, A.Deep Learning, chap. 14 Autoencoders (MIT Press, 2016).

[3] Deng L (2012). The mnist database of handwritten digit images for machine learning research [best of the web]. IEEE Signal Process. Mag..

[4] Cohen, G., Afshar, S., Tapson, J. & Van Schaik, A. Emnist: Extending mnist to handwritten letters. In *2017 International Joint Conference on Neural Networks (IJCNN)*, 2921-2926 (IEEE, 2017).

[5] Yadav, C. & Bottou, L. Cold case: The lost mnist digits.

[6] Uday Prabhu, V. Kannada-mnist: A new handwritten digits dataset for the kannada language. Preprint at https://arxiv.org/abs/1908.01242 (2019).

[7] Xiao, H., Rasul, K. & Vollgraf, R. Fashion-mnist: a novel image dataset for benchmarking machine learning algorithms. Preprint at https://arxiv.org/abs/1708.07747 (2017).

[8] Krizhevsky, A. Learning multiple layers of features from tiny images. Tech. Rep. (2009).

[9] Coates, A., Ng, A. & Lee, H. An analysis of single-layer networks in unsupervised feature learning. In *Proceedings of the fourteenth international conference on artificial intelligence and statistics*, 215-223 (2011).

[10] Yadav, C. & Bottou, L. Cold case: The lost mnist digits. Advances in neural information processing systems 32 (2019).

[11] Northcutt, C. G., Athalye, A. & Mueller, J. Pervasive label errors in test sets destabilize machine learning benchmarks. In *Thirty-fifth Conference on Neural Information Processing Systems Datasets and Benchmarks Track (Round 1)* (2021).

[12] Northcutt C, Jiang L, Chuang I (2021). Confident learning: Estimating uncertainty in dataset labels. J. Artif. Intell. Res..

[13] Jain, S. et al. Visualchexbert: addressing the discrepancy between radiology report labels and image labels. In *Proceedings of the Conference on Health, Inference, and Learning*, 105-115 (2021).

[14] Guss, W. H. & Salakhutdinov, R. On characterizing the capacity of neural networks using algebraic topology. Preprint at https://arxiv.org/abs/1802.04443 (2018).

[15] Goldfarb, D. Understanding deep neural networks using topological data analysis. Preprint at https://arxiv.org/abs/1811.00852 (2018).

[16] Du S (2019). How many samples are needed to estimate a convolutional or recurrent neural network?. stat.

[17] Du, S. & Lee, J. On the power of over-parametrization in neural networks with quadratic activation. In *International conference on machine learning*, 1329-1338 (PMLR, 2018).

[18] Van de Geer, S. A.Applications of empirical process theory, vol. 91 (Cambridge University Press Cambridge, 2000).

[19] Krizhevsky A, Sutskever I, Hinton GE (2012). Imagenet classification with deep convolutional neural networks. Adv. Neural Inf. Process. Syst..

[20] Van Engelen JE, Hoos HH (2020). A survey on semi-supervised learning. Mach. Learn..

[21] Chen, I. Y. et al. Ethical machine learning in healthcare. *Ann Rev. Biomed. Data Sci.* 4, 123–144 (2021). 10.1146/annurev-biodatasci-092820-114757 PMC8362902 34396058

[22] Heo M, Leon AC (2008). Statistical power and sample size requirements for three level hierarchical cluster randomized trials. Biometrics.

[23] Röhmel J (2001). Statistical considerations of fda and cpmp rules for the investigation of new anti-bacterial products. Stat. Med..

[24] Strasak, A. M., Zaman, Q., Pfeiffer, K. P., Göbel, G. & Ulmer, H. Statistical errors in medical research-a review of common pitfalls. *Swiss Med. Wkly.* 137, 44–49 (2007). 10.4414/smw.2007.11587 17299669

[25] Button KS (2013). Power failure: why small sample size undermines the reliability of neuroscience. Nat. Rev. Neurosci..

[26] Carneiro CF, Moulin TC, Macleod MR, Amaral OB (2018). Effect size and statistical power in the rodent fear conditioning literature–a systematic review. PloS one.

[27] Amanatkar HR, Papagiannopoulos B, Grossberg GT (2017). Analysis of recent failures of disease modifying therapies in alzheimer’s disease suggesting a new methodology for future studies. Expert Rev. Neurother..

[28] He J (2019). The practical implementation of artificial intelligence technologies in medicine. Nat. Med..

[29] Balki I (2019). Sample-size determination methodologies for machine learning in medical imaging research: a systematic review. Can. Assoc. Radiologists J..

[30] Dobbin KK, Simon RM (2007). Sample size planning for developing classifiers using high-dimensional dna microarray data. Biostatistics.

[31] DOERSCH C (2016). Tutorial on variational autoencoders. Stat.

[32] Jolicoeur-Martineau, A. Gans beyond divergence minimization. arXiv preprint arXiv:1809.02145 (2018).

[33] Arjovsky, M., Chintala, S. & Bottou, L. Wasserstein generative adversarial networks. In *International conference on machine learning*, 214-223 (PMLR, 2017).

[34] Kermany DS (2018). Identifying medical diagnoses and treatable diseases by image-based deep learning. Cell.

